# An Observational Study on the Association between Migraines and Tension Type Headaches in Patients Diagnosed with Metabolic Syndrome

**DOI:** 10.1155/2013/147065

**Published:** 2013-04-18

**Authors:** Eren Gozke, Muge Unal, Hayriye Engin, Nurbanu Gurbuzer

**Affiliations:** ^1^Department of Neurology, FSM Teaching and Research Hospital, Istanbul, Turkey; ^2^Department of Internal Medicine, FSM Teaching and Research Hospital, Istanbul, Turkey

## Abstract

*Background*. Our aim was to investigate the association between migraine, tension type headache, and metabolic syndrome. *Methods*. Presence of tension type headache and migraine was investigated in 120 patients diagnosed as metabolic syndrome. The severity of the headache was recorded according to the visual analog scale. *Results*. Mean age of the patients was 54.41 ± 11.60 years (range, 29–84 yrs). Diagnoses of tension type headache and migraine without aura were made for 39 (32.5%) and 18 (15%) patients, respectively. Mean age of migraine patients was significantly lower relative to the patients with tension type headache and no headache. Incidence of hypertriglyceridemia was significantly higher in migraine patients when compared with cases tension type headache and without headache. In the tension type headache group, requirement for analgesics decreased as HDL cholesterol levels increased, while need for analgesic drugs increased in line with higher diastolic blood pressures. In the migraine group duration of headache was found to be prolonged with decreasing HDL cholesterol levels. *Conclusion*. In patients presenting with headache, its association with metabolic syndrome should be considered, and the patients should be especially observed with respect to response to analgesic and the presence of hypertension and hyperlipidemia.

## 1. Background

Metabolic syndrome (MetS) is a multifaceted clinical entity in which genetic, hormonal, and environmental factors are involved. Interactions between components of faulty life style such as consumption of excessive sugar and high calorie diet and inadequate exercise are important in MetS. Insulin resistance plays a central role in a series of metabolic disorders. Insulin resistance, microalbuminemia, prothrombotic or proinflammatory conditions alone or in combination with abdominal obesity, dyslipidemia, hypertension, and diabetes, or glucose intolerance are fundamental characteristics of MetS. This syndrome has become a serious public health problem in that it increases risk of development of cardiovascular disease and diabetes with its higher prevalence especially in populations with higher welfare level [1]. 

In recent years, association between metabolic syndrome, obesity, and primary headaches such as migraine and tension type headache has attracted much attention [2–6].

In this study, we aimed to investigate the association between migraine, tension type headache, and MetS and analyze the relationship between components of metabolic syndrome and headache.

## 2. Methods

In 120 patients who were diagnosed as MetS by internist according to National Cholesterol Education Program Adult Treatment Panel III (NCEP ATP-III) criteria [7] were investigated.

The diagnoses of tension type headache and migraine were defined based on International Classification of Headache Disorders-II criteria [8]. All patients were checked for their serum fasting glucose, total cholesterol, HDL and LDL cholesterol, and triglyceride levels. The patients' blood pressures and waist circumferences were measured. The age of onset, frequency, severity, unilateral or bilateral localization, presence of aura, associated symptoms, and response to analgesic use were interrogated. Severity of headache was evaluated to be least severe 1 to most severe 10 according to visual analogue scale (VAS). Patients with concomitant serious diseases (cirrhosis, congestive heart failure, malignancies, chronic diseases, etc.) and cases diagnosed as secondary headache were excluded from the study.

For the statistical analysis of the study data, “Statistical Package for Social Sciences (SPSS) for Windows 17.0” program was used. As descriptive statistical methods mean ± standard deviation was used. In comparison with quantitative data, normality of distribution was assessed using Kolmogorov-Smirnov test. In the comparison of 3 groups, regarding data with normal distribution, one-way ANOVA, and for the comparison of 2 groups Student's *t*-test were used. For data demonstrating abnormal distribution, Kruskal Wallis test was used in the comparisons of 3 groups, while 2 groups were compared using Mann-Whitney *U* test. *Chi-*square test was employed to assess intergroup differences among qualitative data. Interrelationships between quantitative data were assessed using Pearson correlation coefficient. The results were evaluated within 95% confidence interval at a significance level of *P* < 0.05.

## 3. Results

The ages of the patients ranged from 29 to 84 and the mean age was 54.41 ± 11.60. Of the 120 patients diagnosed with the metabolic syndrome, 74.8% (*n* = 89) was females and 25.8% (*n* = 31) was males. According to the International Classification of Headache Disorders-II criteria, 39 (32.5%) of the patients were diagnosed with the episodic tension type headache (ETTH) and 18 (15%) with the migraine without aura (MWOA). Demographic data of the study group are seen in Table 1. Any significant difference was not detected between distribution of genders. Mean age of migraine patients was significantly lower compared with that of patients with ETTH and those without headache.

Distribution of MetS criteria between groups with (MWOA and ETTH) and without pain demonstrated that frequency of hypertriglyceridemia was significantly higher in MWOA cases when compared with patients diagnosed as ETTH and those without pain (Table 2).

Correlations between criteria of metabolic syndrome and characteristics of headache were analyzed, and a statistically significant negative correlation was observed between response to analgesics and HDL cholesterol levels in the ETTH group (*P* < 0.05). HDL cholesterol levels were inversely correlated with patients' need for analgesic drug. In the migraine group a statistically significant negative correlation was found between HDL cholesterol concentrations and duration of pain in years (*P* < 0.05). Duration of headache prolonged with gradually decreasing levels of HDL cholesterol. A statistically significant positive correlation was found between levels of diastolic pressure and analgesic requirements of ETTH group (*P* < 0.05). With increases in diastolic pressure, patients' need for analgesics increased. Visual analogue scale scores were higher in patients with diastolic hypertension (Table 3).

## 4. Discussion

In recent years, metabolic syndrome has gained increasing attention with substantial rates of morbidity and mortality. In the United States, its prevalence in adults is approximately 25% [9]. In Turkey it is seen with an incidence of 28% in men and 40% in women [10]. Increase in the waist circumference is one of the indicators of obesity and also one of the diagnostic markers of metabolic syndrome. Association between obesity and primary headaches was firstly described by Scher et al. [11].

Obesity is a proinflammatory condition with vascular hyperactivity where concentration of inflammatory mediators and plasma CGRP (calcitonin gene-related peptide) increases, while that of adiponectin decreases [3, 4]. These characteristics increase frequency of migraine attacks and induce central sensitization leading to the development of chronic migraine. Many studies investigating the causative relationship between obesity and migraine proved that many inflammatory mediators trigger migraine attacks. Peterlin et al. demonstrated that levels of adiponectin with its lower nociceptive efficacy increased in obesity and contributed to the severity of migraine episodes [12]. As an endocrine gland, adipose tissue produces many inflammatory cytokines. These cytokines (IL-6, TNF-alpha) lead to low-grade inflammation and prothrombotic state. Increase in sympathetic activation is a factor in the development of primary headaches [13–16]. 

Waist circumference is an indicator of accumulated abdominal fat and an indirect marker of insulin resistance and increased proinflammatory cytokine levels [17]. This phenomenon substantiates the hypothesis which assumes that increase in adipose tissue enhances the risk of emergence of migraine attacks and suggestively contributes to the development of other primary headaches like ETTH. In our study 88.8% of the cases in the migraine without aura group and 84.6% of the patients in the ETTH group met the waist circumference criteria of metabolic syndrome.

Hypertriglyceridemia is known as one of the risk factors in migraine [18]. Also in our study, incidence of hypertriglyceridemia in cases with migraine was found to be higher relative to cases with ETTH and those without pain. Negative cholesterol profile (increased LDL and decreased HDL cholesterol) was seen more frequently in cases with migraine. In our study, a positive correlation was found between lower HDL cholesterol levels and duration of pain. A negative correlation between ETTH, HDL cholesterol elevation, and response to analgesics was noteworthy. More severe painful attacks in patients with diastolic hypertension and poor response to analgesics provide important data related to the contribution of hypertension to the severity of pain.

In conclusion, in patients referring with complaints of headache, association between headache and MetS should be considered, and the cases must be followed up especially with respect to the presence of hypertension, hyperlipidemia, and response to analgesic medications.

## Figures and Tables

**Table 1:** Demographic findings of groups.

	ETTH	MWOA	No headache	
	*n* = 39	*n* = 18	*n* = 63
	Mean ± SD	Mean ± SD	Mean ± SD
Age	57.46 ± 11.54	44.83 ± 8.24	55.26 ± 11.18	0.001*

	*n* (%)	*n* (%)	*n* (%)	

Gender				
Male	12 (30.7)	1 (5.5)	18 (28.5)	0.100**
Female	27 (69.3)	17 (94.5)	45 (71.5)	

*Student's *t*-test, **chi-square test.

**Table 2:** The distribution of metabolic syndrome criteria between groups.

	ETTH (*n* = 39)		MWOA (*n* = 18)		No headache (*n* = 63)		*P*
	*n*	%	*n*	%	*n*	%	
Hyperglycemia							
−	10	25.6	7	38.8	20	31.7	0.587**
+	29	74.4	11	61.2	43	68.3	
Hypertriglyceridemia							
−	19	48.7	2	11.1	23	36.5	0.023**
+	20	51.3	16	88.9	40	63.5	
Decreased HDL cholesterol							
−	10	25.6	5	27.7	13	20.6	0.752**
+	29	74.4	13	72.3	50	79.4	
Increased waist circumference							
−	6	15.3	2	11.1	12	19.0	0.704**
+	33	84.7	16	88.9	51	81.0	
Systolic hypertension							
−	12	30.7	7	38.8	29	46.0	0.309**
+	27	69.3	11	61.2	34	54.0	
Diastolic hypertension							
−	16	41.0	8	44.4	33	52.3	0.516**
+	23	59.0	10	55.6	30	47.7	

**Chi-square test.

**Table 3:** Metabolic syndrome criteria and headache features.

	Glucose		Triglyceride		HDL cholesterol		Waist circumference		Systolic hypertension		Diastolic hypertension	
	*P*	*r*	*P*	*r*	*P*	*r*	*P*	*r*	*P*	*r*	*P*	*r*
ETTH												
Duration (year)	0.263	−0.184	0.501	−0.111	0.284	−0.176	0.134	0.244	0.961	0.008	0.550	−0.099
Attack frequency	0.225	0.199	0.197	0.211	0.240	−0.193	0.399	−0.139	0.262	−0.184	0.235	0.195
Severity (VAS)	0.754	−0.052	0.556	−0.097	0.053	0.313	0.819	0.038	0.579	0.092	0.005**	0.441
Response to analgesics	0.339	−0.157	0.963	−0.008	0.033**	−0.343	0.391	0.141	0.272	−0.180	0.039**	−0.332
MWOA												
Duration (year)	0.517	−0.163	0.379	0.220	0.032**	−0.506	0.799	0.065	0.488	0.175	0.891	0.035
Attack frequency	0.802	−0.064	0.406	0.209	0.858	−0.046	0.718	0.091	0.462	0.185	0.290	0.264
Severity (VAS)	0.125	−0.376	0.883	−0.037	0.543	0.153	0.810	0.061	0.683	−0.104	0.825	0.056
Response to analgesics	0.892	−0.035	0.100	−0.400	0.441	0.194	0.069	0.438	0.089	−0.412	0.307	−0.255

**Pearson's correlation.
